# Impact of maternal age on obstetric and neonatal morbidity: a retrospective cohort study

**DOI:** 10.1186/s12884-021-04177-7

**Published:** 2021-10-28

**Authors:** Mélanie Vandekerckhove, Mélanie Guignard, Marie-Sophie Civadier, Alexandra Benachi, Jean Bouyer

**Affiliations:** 1 Service de Gynécologie-Obstétrique, AP-HP, Hôpital Antoine Béclère, 157 rue de la Porte de Trivaux, 92141 Paris, Clamart France; 2 Université Paris-Saclay, 94807 le Kremlin Bicêtre, France; 3 Service du département d’informatique médical, AP-HP, Hôpital Antoine Béclère, 92141 Clamart, France; 4 Université Paris-Saclay, UVSQ, Inserm, CESP, 94807 Villejuif, France

**Keywords:** Age, Advanced maternal age pregnancies, Obstetric morbidity, Neonatal morbidity

## Abstract

**Background:**

Pregnancies in women over 35 years of age are becoming more frequent. The majority of studies point to an age of 35 as a provider of obstetric and neonatal complications. But several confounding factors are not taken into account and this results in contradictory results.

**Methods:**

The objective was to quantify the strength of the association between maternal age and obstetric and neonatal morbidity. This observational study was based on systematic records of 9 years of pregnancies managed in the Obstetrics and Gynaecology Department of Antoine Béclère Hospital, Clamart, France. In all, 24,674 pregnancies were managed at Antoine Béclère Hospital between April 1, 2007 and December 31, 2015, including 23,291 singleton pregnancies. Maternal age was the age at the beginning of pregnancy, taken as a quantitative variable. The main outcome measure was a composite “unfavourable” pregnancy outcome that included miscarriage, induced abortion, in utero foetal death, stillborn or newborn infant weighing under 500 g or delivered before 24 weeks of gestational age. Obstetric and neonatal morbidity comprised hospitalisation during pregnancy for more than 1 day, pre-eclampsia, gestational diabetes requiring hospitalisation, caesarean delivery, preterm birth, small-for-gestational age, and newborn transfer to the paediatric unit or neonatal intensive care unit.

**Results:**

Analyses were conducted among singleton pregnancies (*n* = 23,291) and were adjusted for obesity, assisted reproductive technology and geographical origin of the mother. Unfavourable pregnancy outcome rate tripled with age, from 5% among women aged 25 to 34 to 16% among those over 45. Women over 40 were twice as likely to be hospitalised as those aged 25 to 34. The caesarean section rate reached more than 40% among women over 40 and more than 60% in women over 45. The rate of newborn transfer to paediatric intensive care or a neonatal intensive care unit was doubled in women over 40 and small-for-gestational age was more frequent with age, reaching 34% in women over 45.

**Conclusions:**

The risk of maternal-foetal complications increases steadily with age and is particularly high after 35 years and closer monitoring appears to be necessary. These results provide additional evidence and information for public health decision-makers.

## Key points

Question: How is obstetric and neonatal morbidity associated with maternal age?

Findings: In this study from a French maternity hospital, obstetric and neonatal morbidity increased with maternal age, taking into account parity, obesity, geographical origin and assisted reproductive technology. The increase began at 25 years of age, with no threshold, but with a steeper slope after 35 years. For certain complications in women over 40 years of age, the fold increase could be 2 to 3.

Meaning: Maternal age increases obstetric and neonatal morbidity and these findings are relevant for prospective mothers, the health care team, and public health planning.

## Introduction

In Western countries, pregnancies after 35 years of age (called “late” pregnancies or advanced maternal age pregnancies) are becoming more and more frequent [1, 2], with an average age at first birth of 30.4 years in France in 2016 [3]. Several societal factors may be involved in this trend, such as delay of the first pregnancy beyond 35 years of age to favour the women’s career development [4] or the wish to have a child after a second marriage, a situation more frequent nowadays [5]. Also, advances and expansion in assisted reproductive technology (ART) contribute to the belief that medicine can compensate for the decline in age-related fertility and that pregnancy can be delayed without major difficulties [6, 7].

However, pregnancies after 35 years of age are subject to increased morbidity because of accumulating risks of complications that can occur from the first trimester to the postpartum period [8]. In the first trimester, the rate of miscarriage rises considerably, with several studies showing a three-fold increase in risk for women over 40 compared to those aged 20–34. In addition, the risk of chromosomal abnormalities increases sharply with age [9]. In the second and third trimesters, some maternal and foetal complications increase with age. Most studies [8, 10–31] have found an increased risk of maternal hypertension, pre-eclampsia, gestational diabetes and placenta praevia with age. Neonatal complications include more extreme weight (macrosomia and hypotrophy) and preterm birth (induced or spontaneous). With regard to delivery and the immediate postpartum period, women over 35 years of age have an increased risk of caesarean section [17, 19, 32, 33].

Yet, some reports are not consistent with these results [18, 33, 34], possibly due to the role of confounders such as multiple pregnancies or the use of ART. In addition, maternal age, which is a continuous variable, is often categorised, which may induce misleading thresholds, for instance at 30, 35 and/or 40, resulting in a loss of statistical power in studying the association between age and pregnancy outcome.

The objective of this study was to analyse the relationships between obstetric and neonatal morbidity and maternal age in a large population and without theoretical thresholds.

## Methods

### Study design

The study covered all pregnancies between April 1, 2007 and December 31, 2015 managed in the Antoine Béclère Hospital Obstetrics Department, which is a level III maternity unit, i.e. is allowed to manage all types of pregnancies and has access to a neonatal intensive care unit. The analyses were limited to singleton pregnancies.

### Studied variables

The data were recorded during each pregnancy as part of the usual computerised medical monitoring conducted in the Antoine Béclère Hospital. They were extracted, managed and anonymised by the Clinical Research Unit.

Maternal age was the age at the beginning of pregnancy, taken as a quantitative variable. The general characteristics of the women were: obesity (body mass index ≥30), multiparity, ART use and geographical origin (Europe, North Africa (Maghreb), sub-Saharan Africa (non-Maghreb), Asia, other).

As for pregnancy complications, we first considered a composite “unfavourable” pregnancy outcome defined as a pregnancy not ending in a live birth (miscarriage, induced abortion, in utero foetal death, stillborn) or ending in the birth of a newborn weighing under 500 g or delivered before 24 weeks of gestational age (GA). Voluntary interruption of pregnancy was excluded. Among other pregnancies (i.e. that ended in the birth of a newborn ≥500 g and at GA ≥ 24 weeks), we considered the following obstetric complications: hospitalisation during pregnancy for more than 1 day, pre-eclampsia, gestational diabetes requiring hospitalisation, caesarean section. Neonatal morbidity comprised: preterm birth (delivery before 37 weeks), small-for-gestational age (SGA, birth weight below the 10th percentile for sex and GA according to French curves [25]), and newborn transfer to the paediatric unit or neonatal intensive care unit.

### Analysis

Analyses of the women’s characteristics and adverse pregnancy outcomes were conducted for the entire sample of singleton pregnancies, while analyses of obstetric and neonatal morbidity were conducted for pregnancies that ended in delivery of a live newborn over 500 g and at GA ≥ 24 weeks.

The relationship between age and obstetric morbidity was analysed and modelled with logistic regression, keeping age in quantitative form and using fractional polynomials that give an optimal data fit [26]. This method allows the associations between maternal age and complications to be presented as easy-to-understand curves with their 95% confidence interval (CI). Results were also displayed in 5 age classes to enable comparison with other studies: < 25; 25–34; 35–39; 40–44; ≥45. The predicted values of the percentages of complication by class and their 95% CI were derived from the previous modelling. All the analyses were adjusted for obesity, ART use and geographical origin. The statistical analyses were performed with Stata 15 [27].

### Patient and public involvement

Patients were not involved in setting the research questions or planning the study. The investigators did not know the identity of the study participants.

### Ethical approval

The study was approved by the CNIL (French Data Protection Authority) and given the number 2118329 v 0. The local institutional review board approved the study (Research Ethics Committee in Obstetrics and Gynaecology. CEROG 2018-OBST-0402).

## Results

Between 2007 and 2015, 24,674 pregnancies were recorded. Mean maternal age was 30.7, range [13–54] and interquartile interval [27–34]. Table 1 displays the age distribution and the general characteristics of singleton pregnant women.

**Table 1:** Characteristics of the study population (singleton pregnancies)

Total 23,291		< 25	25–34	35–39	40–44	≥ 45	
N (%)		2834 (12.2)	14,815 (63.6)	4480 (19.2)	1085 (4.7)	77 (0.33)	
Obesity (%)		10.1	9.1	11.4	14.7	7.0	
ART (%)		0.5	3.5	8.5	15.4	44.2	
Geographical origin Europe (%)		49.2	64.	65.0	65.2	63.5	
pregnancies with “unfavourable” outcome (%)		5.9	5.1	6.9	9.3	15.4	
	in utero *foetal death*	0.6	0.9	1.2	.1.5	2	
	*medically induced abortion*	2.6	3.7	4.5	5.2	6.3	

**Fig. 1:**
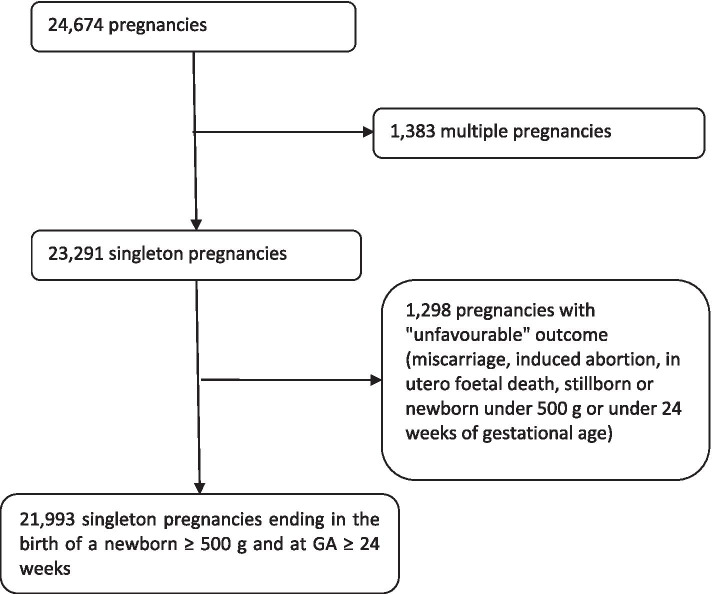
Flow Chart

Of the 23,291 singleton pregnancies, 21,993 resulted in delivery of a live newborn over 500 g and at GA ≥ 24 weeks (Fig. 1). Analyses of obstetric and neonatal morbidity were performed for pregnancies that ended with the delivery of a live newborn of more than 500 g and at a GA ≥ 24 weeks.

**Fig. 2:**
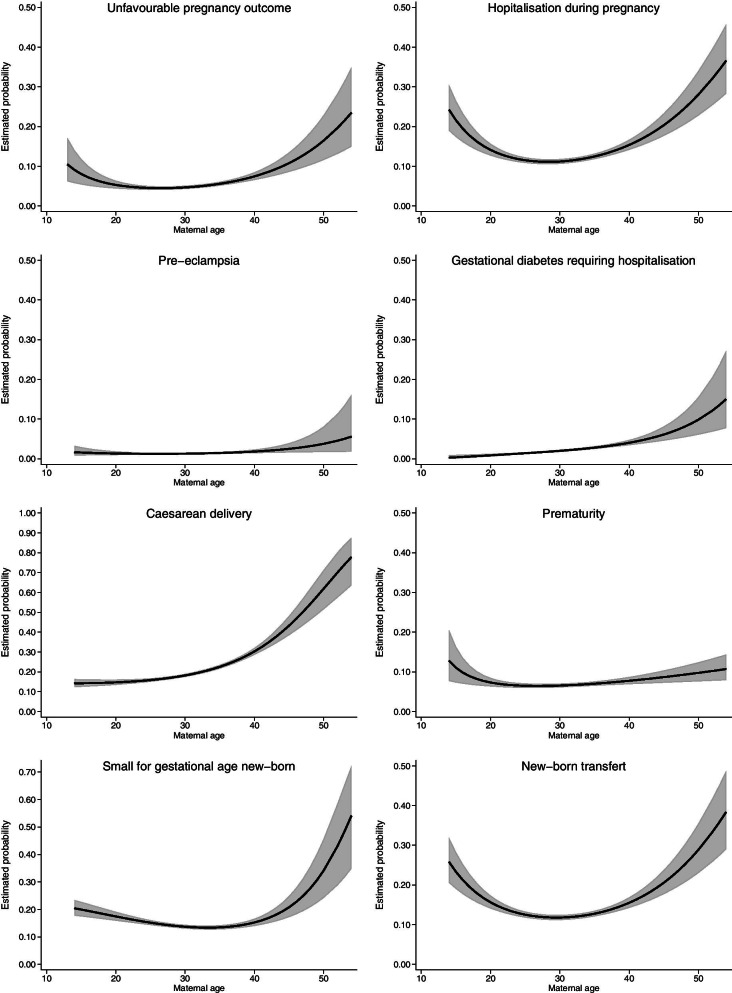
Relationship between maternal age and obstetric and neonatal morbidity. Estimated probabilities (solid line; 95% CI in grey) were provided by modelling the relationship between maternal age and outcome with logistic regression and fractional polynomials (see "Analysis" section). All relationships are adjusted for obesity, assisted reproductive technology and geographical origin

The shapes of the relationships between maternal age and obstetric or neonatal morbidity adjusted for obesity, parity, ART use and geographical origin are provided in Fig. 2 and the predicted percentages and 95% CI by age classes are displayed in Table 2.

**Table 2:** Estimated probabilities and 95% CI of obstetric and neonatal morbidity according to maternal age adjusted for: obesity, assisted reproductive technology and geographical origin. Probabilities were estimated by modelling the relationship between maternal age and outcome (see Fig. 2)

		Maternal age				*P* ^1^
	< 25	25–34	35–39	40–44	≥ 45	
Unfavourable pregnancy outcome	0.06 [0.05–0.08]	0.05 [0.04–0.05]	0.07 [0.06–0.07]	0.09 [0.08–0.11]	0.16 [0.12–0.23]	< 0.001
Hospitalisation during pregnancy ^a^	0.16 [0.14–0.19]	0.11 [0.11–0.12]	0.14 [0.13–0.15]	0.18 [0.16–0.20]	0.28 [0.23–0.34]	< 0.001
Pre-eclampsia	0.01 [0.01–0.02]	0.01 [0.01–0.02]	0.02 [0.01–0.02]	0.02 [0.02–0.03]	0.04 [0.02–0.08]	0.02
Gestational diabetes ^b^	0.01 [0.00–0.01]	0.02 [0.02–0.02]	0.03 [0.03–0.04]	0.05 [0.04–0.06]	0.10 [0.06–0.16]	< 0.001
Caesarean delivery	0.14 [0.13–0.16]	0.18 [0.18–0.19]	0.27 [0.25–0.28]	0.37 [0.34–0.41]	0.62 [0.52–0.71]	< 0.001
Preterm birth	0.08 [0.07–0.10]	0.07 [0.06–0.07]	0.07 [0.07–0.08]	0.08 [0.07–0.10]	0.10 [0.08–0.12]	0.01
Newborn small-for-gestational age	0.18 [0.17–0.21]	0.14 [0.13–0.14]	0.14 [0.13–0.15]	0.18 [0.16–0.21]	0.34 [0.24–0.46]	< 0.001
Newborn transfer ^c^	0.18 [0.16–0.21]	0.12 [0.11–0.12]	0.14 [0.13–0.15]	0.18 [0.16–0.20]	0.29 [0.23–0.36]	< 0.001

^1^ test of the association with age as a continuous variable

^a^ more than 1 day

^b^ requiring hospitalisation

^c^ in paediatric unit or neonatal intensive care unit

All complications increased continuously and significantly beyond the age of 25 or 30. Some maternal age relationships have a U-shape, indicating increased risk of complications for younger as well as for older women: hospitalisation during pregnancy, newborn transfer, SGA and to a lesser extent unfavourable pregnancy outcome.

Some results deserve to be highlighted. Unfavourable pregnancy outcome rate tripled with age, from 5% among women aged 25 to 34 to 16% among those over 45. Overall, women over 40 were twice as likely to be hospitalised as those aged 25 to 34. The caesarean section rate reached more than 40% among women over 40 and more than 60% in women over 45. This increase was mainly due to planned caesarean section, since the rate of emergency caesarean section remained steady with maternal age at a value of around 12% (data not shown).

The rate of newborn transfers to the paediatric unit or neonatal intensive care unit doubled in women over 40 and SGA was much more frequent with age, reaching 34% in women over 45.

## Discussion

### Statement of principal findings

There was a continuous increase in obstetric and neonatal morbidity with maternal age after 30 years. Although there was no “threshold”, the increase became more marked after 35 years. In addition, an age below 20 years is also more associated with obstetrical complications.

### Strengths and weaknesses of the study

The main strength of our study is its large sample size and the fact that the management of pregnancies at the Antoine Béclère Hospital remained the same throughout the study, although it is a hospital-based study. In addition, the data were recorded as time goes by as part of the pregnancy medical follow-up, which ensures that there is no bias related to the objectives of the present study. However, some data that were not collected routinely were missing, such as tobacco consumption, alcohol consumption, and socioeconomic level. The study sample may be not representative of the French population of pregnant women, but this is unlikely to bias the association between maternal age and pregnancy complications. In addition, we verified that the main characteristics of the study sample were similar to those of the 2016 French national perinatal survey [3]: pregnancy rate beyond 35 years and hospitalisation rate during pregnancy, for instance.

### Strengths and weaknesses in relation to other studies: important differences in results

This study takes into account potential confounding factors such as multiple pregnancies and the use of ART. It shows that there is no threshold, but rather a continuous increase in the risk of complications with maternal age after 30 years. The results confirm those of previous studies [9, 17, 19, 34] with great accuracy and in another population, which reinforces their validity. They also show that there is an “optimal gap” between about 20 and 35 years of age that minimises obstetric and neonatal complication rates.

### Meaning of the study: possible explanations and implications for clinicians and policy makers

Since the main confounding factors have been taken into account, the results of this study provide additional evidence of the specific role of maternal age in increasing pregnancy complications: unfavourable outcome, hospitalisation, pre-eclampsia, gestational diabetes, caesarean section, SGA babies and newborn transfers. This justifies increased surveillance, especially from the third trimester of pregnancy with, for example, more frequent medical appointments (consultation and ultrasound) and foetal monitoring.

Delayed childbearing therefore exposes women to an increase in obstetric and neonatal complications as described in the article. This tendency to delay childbearing in Western societies is multifactorial. The evolution of our society is marked in particular by an increase in the level of education, with a postponement of entry into working life. Indeed, 68% of young people born between 1987 and 1991 have a bachelor’s degree, compared with 44% of those born between 1967 and 1972 [35]. At the same time, the higher the level of education, the later the age of first childbearing [36], as women wish to develop their professional careers before starting a first pregnancy.

Secondly, great progress has been made in medically assisted reproduction in terms of technique and effectiveness. Media coverage of these techniques has probably contributed to the trivialisation of medically assisted reproduction and has promoted the concept of easily achievable late pregnancy as a possible recourse in prioritising one’s career.

Thirdly, there is an increase in the number of older multiparous women linked to changes in the so-called traditional family with increasing divorce and the creation of blended families [37].

The basic issue seems to be to find a balance between the current social autonomy and empowerment of women and the “biological limits” that natural evolution has not yet changed. Perhaps a more family-friendly social policy without giving up professional career development could help motivate couples to start a family earlier. In parallel, awareness-raising campaigns on the decline in fertility from the age of 30 and the increase in maternal and neonatal complications seem to be indispensable.

### Unanswered questions and future research

Some articles, such as those of Guilbaud et al. [28] and Henne et al. [29], show that oocyte donation, regardless of age, is a factor in its own right in obstetric complications. Autologous oocyte preservation for age-related fertility decline is performed in many countries and recommendations on authorised age limits for reimplantation are still lacking. Due to a small sample size and a lack of information, particularly on in vitro fertilisation modalities, we could not study in our sample the use of in vitro fertilisation and/or oocyte donation in people over 45 years of age. Studies are needed to evaluate whether the relationship between maternal age and morbidity is reproducible if oocyte age differs from the patient’s age at the time of pregnancy.

## Conclusion

Our study shows a significant association of obstetric morbidity with age. There is a steady and continuous increase with age in the rate of pre-eclampsia, gestational diabetes, hospitalisation, prematurity and caesarean section. This increase is greater after the age of 35. However, pregnancies beyond the age of 35 are increasing and it seems necessary to inform women of the risks involved in postponing motherhood.

## Data Availability

The data of the study cannot be shared publicly as they contain sensitive patient information and are the property of AP-HP (Paris Public Hospitals). Consultation of the data by other interested researchers may be considered by AP-HP, subject to prior determination of the terms and conditions of such consultation and in respect of compliance with the applicable French and European regulations. Requests should be addressed to the Delegation for Clinical Research and Innovation (DRCI) at secretariat-direction.drc@aphp.fr.
